# Biliary Tract Cancers: Molecular Heterogeneity and New Treatment Options

**DOI:** 10.3390/cancers12113370

**Published:** 2020-11-13

**Authors:** Nicola Personeni, Ana Lleo, Tiziana Pressiani, Francesca Colapietro, Mark Robert Openshaw, Chara Stavraka, Athanasios Pouptsis, David James Pinato, Lorenza Rimassa

**Affiliations:** 1 Medical Oncology and Hematology Unit, Humanitas Cancer Center, Humanitas Clinical and Research Center-IRCCS, Rozzano, 20089 Milan, Italy; nicola.personeni@hunimed.eu (N.P.); tiziana.pressiani@cancercenter.humanitas.it (T.P.); 2 Department of Biomedical Sciences, Humanitas University, Pieve Emanuele, 20090 Milan, Italy; ana.lleo@humanitas.it (A.L.); francesca.colapietro@humanitas.it (F.C.); 3 Internal Medicine Center, Humanitas Clinical and Research Center-IRCCS, Rozzano, 20089 Milan, Italy; 4 Department of Surgery & Cancer, Imperial College London, Hammersmith Hospital, London W120HS, UK; mro6@leicester.ac.uk (M.R.O.); david.pinato@imperial.ac.uk (D.J.P.); 5 Department of Medical Oncology, Guy’s and St Thomas’ NHS Foundation Trust, Great Maze Pond, London SE1 9RT, UK; chara.stavraka@kcl.ac.uk; 6 Department of Medical Oncology, “Euromedica” General Clinic, 54645 Thessaloniki, Greece; Ath.pouptsis@oncomedicare.com

**Keywords:** biliary tract cancer, cholangiocarcinoma, molecular characterization, tumor heterogeneity, FGFR, IDH, targeted agents

## Abstract

**Simple Summary:**

Incidence of biliary tract cancer is increasing, and patients are frequently diagnosed with unresectable or metastatic disease, when therapeutic options are limited. Due to these reasons, prognosis remains poor and new systemic treatment options are urgently needed. This article reviews the new available data on molecular heterogeneity of biliary tract cancer and especially intrahepatic cholangiocarcinoma and the novel therapeutic strategies offered by the improved knowledge of the biology of this disease. For these reasons, this topic is of relevant interest for the oncology and hepatology community.

**Abstract:**

Most patients with biliary tract cancer (BTC) are diagnosed with advanced disease, relapse rates are high in those undergoing surgery and prognosis remains poor, while the incidence is increasing. Treatment options are limited, and chemotherapy is still the standard of care in both adjuvant and advanced disease setting. In recent years, different subtypes of BTC have been defined depending on the anatomical location and genetic and/or epigenetic aberrations. Especially for intrahepatic cholangiocarcinoma (iCCA) novel therapeutic targets have been identified, including fibroblast growth factor receptor 2 gene fusions and isocitrate dehydrogenase 1 and 2 mutations, with molecularly targeted agents having shown evidence of activity in this subgroup of patients. Additionally, other pathways are being evaluated in both iCCA and other subtypes of BTC, alongside targeting of the immune microenvironment. The growing knowledge of BTC biology and molecular heterogeneity has paved the way for the development of new therapeutic approaches that will completely change the treatment paradigm for this disease in the near future. This review provides an overview of the molecular heterogeneity of BTC and summarizes new targets and emerging therapies in development. We also discuss resistance mechanisms, open issues, and future perspectives in the management of BTC.

## 1. Introduction

Biliary tract cancers (BTC) represent a heterogeneous group of cancers arising from the bile ducts. Based on anatomical location, BTC are classified into intrahepatic cholangiocarcinoma (iCCA), perihilar CCA (pCCA), distal CCA (dCCA), and gallbladder cancer (GBC) [1]. They represent 3% of all gastrointestinal neoplasms in adults, the most frequent anatomic subtype originates from the extrahepatic biliary tract [2], but their incidence is increasing worldwide, mainly due to iCCA [3,4,5], which is the second most common type of primary liver cancer, responsible for 20% of liver-related deaths [6]. Anatomic subtypes have different risk factors, clinical presentations, molecular alterations, and prognosis [7,8,9]. Risk factors for iCCA include primary sclerosing cholangitis, Caroli’s disease, lithiasis, liver flukes, viral hepatitis, cirrhosis, obesity, and diabetes [7,10,11]. Given the increasing incidence and prevalence of metabolic syndrome, it is estimated that also the incidence of iCCA will increase in the coming years, [12,13]. However, about 50% of cases have no identifiable risk factors and there are no clinically applicable biomarkers for early diagnosis. As a result, BTCs are frequently diagnosed at an advanced stage when treatment options are limited and prognosis remains poor with a 5-year survival of about 5–15% [14,15]. Furthermore, up to 20% of cancers of unknown primary site can be reconducted to a biliary tract origin based on the molecular profile, further highlighting the challenges of an early diagnosis [16].

**Figure 1:**
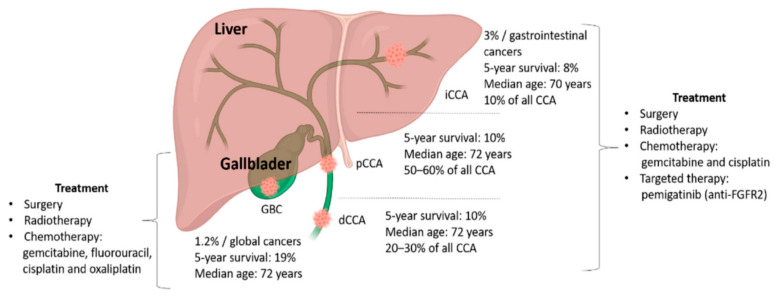
Schematic representation of the anatomic site, demographic information, and treatment options for biliary tract cancers (BTC). Abbreviations: dCCA: Distal cholangiocarcinoma (CCA); GBC: Gallbladder cancer; iCCA: Intrahepatic cholangiocarcinoma; pCCA: Perihilar CCA. From Malenica, I. et al. Cancers (Basel) 2020 [23].

In the adjuvant setting, a benefit in relapse-free survival and overall survival (OS) has been shown with capecitabine compared to observation in the randomized phase III BILCAP trial [17] and this treatment has been included in international guidelines, as well as radiation therapy for patients with extrahepatic cholangiocarcinoma (eCCA) or GBC and a microscopically positive surgical margin resection (R1 resection) [18]. In advanced disease, the combination of cisplatin and gemcitabine still remains the only standard first-line treatment option based on the positive results of the phase III ABC-02 study reported a decade ago [19,20,21]. Recently, the phase III ABC-06 study demonstrated the advantage of modified FOLFOX (5-fluorouracil, folinic acid, oxaliplatin) as a second-line therapy compared to active symptom control [22] (Figure 1).

Considering this scenario, a better understanding of BTC characteristics, pathogenesis, and molecular heterogeneity is urgently needed to develop strategies for early diagnosis, identify new biomarkers, and define new patient-tailored therapeutic approaches with the overarching aim of improving patient outcomes [24,25,26,27,28]. In particular, the BTC phenotype is related to the genetic and epigenetic alterations in tumor cells and is also influenced by the molecular cross-talk between the neoplastic cells and the surrounding microenvironment [1,11]. New molecular alterations have been identified in BTC by recent genetic studies, which have provided new insights into the pathogenic mechanisms of this disease and the signaling pathways that drive its progression. In particular, a large comprehensive study on the molecular profile of BTC has shown that nearly 40% of the patients have potentially targetable genetic alterations [8] also showing clear differences between the different subtypes of BTC. The paradigm shift towards a personalized molecular therapy opened up new options and brought new hope for patients with BTC. Importantly, the emergence of targeted therapies is rapidly changing the treatment approach for BTC, especially for iCCA. Recent positive phase II and III trials of new targeted therapies [29,30,31,32] have been published and the United States Food and Drug Administration (FDA) has approved pemigatinib, a fibroblast growth factor receptor (FGFR) inhibitor, for patients with advanced BTC with *FGFR2* gene fusions, previously treated with the standard of care, making pemigatinib the first targeted agent approved for patients with BTC [30]. However, genetic or epigenetic aberrations have only been identified in small subgroups, and precision medicine remains an important unmet need for most of the patients with advanced BTC [15]. For all these reasons, patients with BTC should be evaluated in referral centers where dedicated multidisciplinary teams along with a strong collaboration between researchers and clinicians enable them to receive optimal care, have access to ongoing clinical trials and ultimately improve their prognosis [15,33].

In this review, we will provide a comprehensive overview of how the molecular heterogeneity of BTC is rapidly reshaping the therapeutic landscape in this therapeutically deprived disease area, focusing in particular on patients with advanced disease. Immunological characterization of BTC and potential immunotherapeutic strategies have been reviewed elsewhere [1].

## 2. Genomic Heterogeneity of BTC

**Figure 2:**
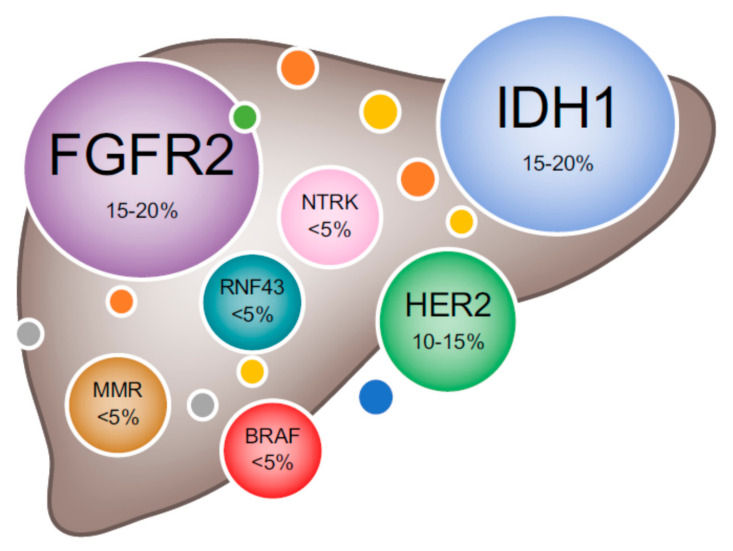
Schematic overview of therapeutically actionable drivers in biliary tract cancers. Abbreviations: FGFR2: Fibroblast growth factor receptor 2; HER2: Human epidermal growth factor receptor 2; IDH1: Isocitrate dehydrogenase 1; NTRK: Neurotrophic tyrosine receptor kinase; MMR: mismatch repair deficiency. Reproduced with permission from Elsevier, from Lamarca, A. et al. J. Hepatol. 2020 [15].

In-depth sequencing of BTC has highlighted the genomic complexity of cholangiocarcinoma (CCA), and importantly, the fact that genetic driver mutations vary between different subtypes of CCA [15] (Figure 2).

### 2.1. Intrahepatic Cholangiocarcinoma

Deep sequencing studies have improved our understanding of the genomic complexity in iCCA and have identified the main targetable genetic alterations in the form of isocitrate dehydrogenase (*IDH*) and *FGFR*. Interestingly, the predominant genomic alterations in CCA are associated with epigenetic processes; 10–20% of iCCA tumors present mutations in *IDH*, with *IDH1* and *IDH2* being the ones of relevance in cancer, due to their involvement in cell metabolism [34]. Further, Nakamura described for the first time the *FGFR2* fusion genes, exclusively detected in iCCA [8]. *FGFR2* rearrangement is the most common type of *FGFR* aberration with prevalence 14%–23% and occurring almost invariably in iCCA [35,36,37]. BICC1 is the most frequent FGFR2 rearrangement partner (29.7%) [38] whereas, confirming findings from prior studies of whole-genome and targeted exon sequencing of iCCA [8,39], FGR2 translocation has been reported as mutually exclusive with *IDH1* mutations [35].

Research by Sia and colleagues showed that iCCA tumors can be classified into two groups (i.e., inflammation and proliferation), with distinct characteristics, activated genes, and clinical outcome [40]. The inflammation class (38% of iCCAs) presents activated inflammatory signaling pathways, with overexpression of cytokines, and STAT3 activation. On the other side, the proliferation class (62%) is characterized by activation of oncogenic signaling pathways (i.e., RAS, MAPK, and MET), mutations in *KRAS* and *BRAF*.

Importantly, it has also been indicated that mutations tend to cluster together and that tumors with *IDH* mutations have been found to have specific mRNA, copy number, and increased methylation of the *ARID1A* promoter. In contrast, FGFR2-positive tumors seem to associate with BAP1 mutations [39]. In addition, molecular iCCA findings seem to vary depending on etiology, as demonstrated by a recent study that highlights a strong genetic heterogeneity between Western and Asian patients [41].

### 2.2. Extrahepatic Cholangiocarcinoma

Therapeutic approaches for eCCA are limited. The treatment of choice for early stages is surgical resection, however the risk of recurrence is high and the majority of cases present when the disease is unresectable. Unfortunately, no molecular targeted therapies have been currently identified [32] and the current standard of care offers a small benefit [19]. Previous obstacles to performing a molecular characterization of eCCA included the low number of samples analyzed in international cancer genome projects and the heterogeneity of the cohorts including a wide range of other subtypes of CCA, mainly iCCA [8].

Before the consensus in CCA subtype classification was accepted, a small cohort of 40 Caucasian patients with eCCA was analyzed and a SNP at a position 825 C > T in GNB3 gene was identify as having a prognostic role [42]. Although of small clinical relevance, this was the first evidence of genomic heterogeneity in eCCA.

Until now, the main targetable findings of eCCA rely on the HER gene family in 5–9% of cases [43], also fusion aberrances ATP1B-PRKACA and ATP1B-PRKACB were described to be exclusively present in eCCA [8]. Recently, a large study using integrative molecular analysis of 189 eCCA found that *KRAS, TP53, ARID1A* and *SMAD4* are the most prevalently mutated genes, with near 25% of tumors displaying at least one actionable genomic alteration (*EGFR, ERBB2, BRCA1/2, IDH1/2, CDK4, BRAF, PIK3CA, MDM2* and *NRAS*) [44]. Further, based on the transcriptomic data, the authors identified four molecular groups (identified as Metabolic, Proliferation, Mesenchymal, and Immune) with distinct oncogenic signatures and histological subtypes. Importantly this work provides a comprehensive picture of eCCA as a distinct molecular entity and opens the door for a targeted treatment approach.

### 2.3. Gallbladder Cancer

Until now, little knowledge has come from Genome Wide Association Studies (GWAS) in GBC. Indeed, genetic variants associated with increased risk of GBC have been explored using small cohorts and conclusive determinations of relative risk will require larger studies. Further, most of the available studies focus on one candidate gene.

A recent GWAS focusing on gallstone disease, the main risk factor for GBC, with a subgroup analysis of GBC in the presence of gallstone disease, identified *ABCG8* and *TRAF3* genes as significantly associated with gallstone disease and GBC in Latinos [45]. The extensive whole-exome and transcriptome (RNASeq) sequencing analysis of 29 GBC in Japanese patients found that 35.8% of them present mutations in the ERBB family of proteins (including their downstream genes) [8]. Importantly, it might be targeted with novel tyrosine kinase inhibitors and monoclonal antibody strategies [46,47]. Moreover, a case-control GWAS of GBC and controls of Indian descent showed significant associations for *ABCB1* and *ABCB4* genes. Importantly, *ABCB1* and *ABCB4* genes encode transporters for phospholipids across the biliary epithelium, which might function to modulate bile solubility and therefore the propensity for gallstone formation [48].

Finally, the presence of activating mutations in *PIK3CA* and *HER2* aberrations have also been identified in GBC, while *IDH* mutations are absent in this type of cancer [43].

## 3. Epigenetics

Given the substantial gap between current knowledge on genetic alterations that affect BTC [49], unfavorable clinical outcomes, and subsets of tumors harboring significantly different transcriptomic profiles [24], the attention is currently turning on epigenetic alterations that serve as potential pro-oncogenic lesions leading to disparate patterns of DNA methylation, chromatin remodeling or aberrant expression of non-coding RNA. Much of this information is deriving from current investigations on iCCA.

In contrast to canonical oncogene mutations that drive signaling deregulation within a single pathway, mutant or aberrantly expressed epigenetic regulators generally portend genome-wide perturbations that result into different transcriptomic patters.

Altered DNA methylation is one of the earliest molecular alterations that characterize tumor development [50]. In particular, hypermethylation occurring in tumor suppressor promoter is a key determinant for the transcriptional inactivation of a gene. Irrespective of the intrahepatic or extrahepatic site, CCA typically exhibit DNA promoter hypermethylation that is reported in at least 85% of CCA cases [51].

Moreover, recent investigations have pointed out recurrent inactivating mutations or deletions in multiple chromatin-remodeling genes broadly acting as tumor suppressors, such as *ARID1A*, *PBRM1* and *BAP1* [52]. Nonsynonymous mutations in chromatin remodelers randomly occur throughout the gene in the absence of a specific hotspot and they are causative of a loss of function with subsequent malignant transformation. In contrast, the non-random binomial distribution of hypermethylation patterns suggests the underpinning of specific mechanisms [53] still to be fully elucidated.

ARID1A, which retains a DNA binding activity, and PBRM1, which binds to histones, belong to the SWI/SNF chromatin-remodeling complex, whose expression is lost in advanced CCA, but it is still detectable in pre-malignant lesions. Importantly, SWI/SNF mediates the repositioning of nucleosomes to allow an efficient DNA repair, while regulating transcription and DNA replication [54]. Since mutations inducing loss of function in ARID1A and BAP1 may also inhibit double-strand break repair (DSR) [55], an increased sensitivity to poly ADP ribose polymerase inhibitors (PARPi) has been postulated [56]. A phase II clinical trial testing the PARPi niraparib is underway (NCT03207347) in CCA and solid tumors carrying mutations of genes that regulate the DSR, including ARID1A and BAP1.

Dysregulated expression of epigenetic regulators and histone post-translational modifications represent additional mechanisms mediating epigenetic perturbations in CCA. In particular, recent investigations underscore that the overexpression of EZH2, a histone methyltransferase associated with transcriptional repression, associates with poor prognosis in both intrahepatic and extrahepatic CCA [57].

*IDH* mutations alone have been associated with a hypermethylator phenotype at both the DNA and histone level [58], with nearly 50% of the hypermethylated genes corresponding to silenced genes identified in glioblastomas [59]. Whereas *IDH1* mutations are more frequent than *IDH2* mutations [60], both mutations are mutually exclusive and tend to appear more frequently in recurrent iCCA (Figure 2). In contrast, they are virtually absent in pCCA and dCCA [61].

The neomorphic IDH enzymes are responsible for the conversion of alpha-ketoglutarate (the end product of IDH enzymes under normal conditions) to the oncometabolite 2-hydroxyglutarate. This eventually contributes to the raising levels of histone and DNA methylation through the inhibition of demethylases and hydroxylases functions [62]. Given the enhanced expression of epithelial-to-mesenchymal transition (EMT) traits [63] observed in *IDH* mutant tumors, putative genes targeted by such genome-wide epigenetic perturbations are felt to play a role in cell differentiation [64].

Differences across Eastern and Western cohorts have been reported in terms of frequency of *IDH* mutations [65], suggesting the impact of environmental and etiological factors represented by prevalence of liver fluke infection and hepatitis. In fact, significant differences between mechanisms that drive aberrant DNA hypermethylation differentiate liver flukes-related iCCA from other IDH1/2 and BAP1 mutant iCCA [66]. Whereas liver flukes are associated with CpG island hypermethylation, iCCA carrying *IDH1/2* and *BAP1* mutations show a prevalent hypermethylation of the CpG shores (the regions immediately flanking CpG islands), implying that somatic mutations in non-fluke-related iCCA determine the downstream hypermethylation patterns. Of note, a better prognosis has been reported for *IDH*-mutant iCCA when compared to fluke-related iCCA [66].

*IDH* mutant CCA display significantly decreased chromatin modifier expression, as well as increased mitochondrial gene expression [64]. Consistently, *IDH* mutants hypermethylate and silence the *ARID1A* promoter [39], which may contribute to an overall lowered chromatin modifier expression. This is in line with an epigenomic “snowball effect” [54] whose existence has been postulated examining the sequence that first involves mutant *IDH* (acting as an oncogene) and downregulated chromatin modifiers (acting as tumor suppressors) thereafter.

Besides protein coding genes, accumulating data suggest that epigenetic alterations in CCA can be related to aberrant expression of non-coding RNAs including several families of microRNAs (miRNAs). miRNAs have been shown to modify the expression of several genes regulating cell proliferation and migration, EMT and cell invasion [67].

However, independent studies in CCA have reported an altered expression for only few miRNAs.

## 4. Systemic Treatment for Advanced BTC

### 4.1. Chemotherapy

Since the 90s chemotherapy demonstrated an improvement in OS and quality of life compared to best supportive care resulting in an improvement in median OS of 6 vs. 2.5 months (*p* < 0.01) for unresectable, metastatic or recurrent BTC [68]. Many agents have been tested either alone or in combination in different studies. The results were collected in 2007 in a pooled analysis of 104 clinical trials, including 2810 patients, which showed a better outcome for doublet chemotherapy compared with monotherapy, particularly for gemcitabine in combination with cisplatin or oxaliplatin [69]. Gemcitabine, platinum compounds and fluoropyrimidines represent the most active agents for the treatment of advanced BTC [70].

In 2010 the positive results of the ABC-02 randomized phase III study established the doublet cisplatin and gemcitabine as first-line standard of care for advanced BTC, showing a statistically significant OS and progression-free survival (PFS) advantage in all the anatomical subgroups with the doublet regimen over single-agent gemcitabine (OS 11.7 vs. 8.1 months; HR 0.64; 95% CI 0.52–0.80; *p* < 0.001—PFS 8.0 vs. 5.0 months; HR 0.63; 95% CI 0.51–0.77; *p* < 0.001), at the expense of higher rate of toxic effects, mainly hematological [19]. The results were confirmed in the Japanese randomized phase II BT-22 study that showed a median OS of 11.2 months for the combination vs. 7.7 months for gemcitabine monotherapy (HR 0.69; 95% CI 0.42–1.13; *p* = 0.14) and a PFS of 5.8 vs. 3.7 months (HR 0.66; 95% CI 0.41–1.05; *p* = 0.08) [20]. These data were also confirmed in a meta-analysis of the two trials [21].

A post-hoc analysis of patient data collected from three first-line trials (namely, ABC-01, ABC-02, ABC-03) was performed to explore the outcome of patients with iCCA treated with cisplatin and gemcitabine, showing prolonged OS and PFS compared with patients with non-iCCA (OS 15.4 months, 95% CI 11.1–17.9; PFS 8.4 months, 95% CI 5.9–8.9). Furthermore, a similar trend in OS, even if not statistically significant, was confirmed for patients with liver-only iCCA (HR 0.65; 95% CI 0.36–1.19; *p* = 0.16) [9]. In addition, retrospective data support the combination of cisplatin and gemcitabine also in fit patients with jaundice related to biliary tract obstruction but not in those with jaundice due to extensive liver involvement [71]. The replacement of cisplatin by oxaliplatin in the GEMOX regimen usually represents an alternative as first-line treatment in patient unfit for cisplatin based on the results of a non-randomized phase II study [72].

In the second-line setting, the phase III randomized ABC-06 study demonstrated the advantage of modified FOLFOX compared to active symptom control, following failure of first-line cisplatin and gemcitabine [22]. The study showed a significantly improvement of OS (6.2 vs. 5.3 months, HR 0.69; 95% CI 0.50–0.97; *p* = 0.031) regardless of response to prior cisplatin.

A Japanese phase III randomized trial (FUGA-BT) explored the combination of gemcitabine plus S1 versus gemcitabine and cisplatin and demonstrated that the new combination was non-inferior to the standard of care, with a median OS of 15.1 and 13.4 months, respectively (HR 0.945; 90% CI 0.78–1.15; *p* = 0.046 for non-inferiority), and a good tolerability profile [73].

To improve the efficacy of systemic treatment, clinical trials adding a third drug to the reference first-line doublet were conducted. In a phase II trial treatment with nab-paclitaxel plus cisplatin and gemcitabine showed a median PFS of 11.8 months (95% CI 6.0–15.6) and median OS of 19.2 months (95% CI 13.2–not estimable) with an 84% disease control rate (DCR) [74]. A Japanese phase III study (KHBO1401-MITSUBA) demonstrated prolonged OS in 246 patients adding S1 to cisplatin and gemcitabine. This study showed an advantage for the triplet arm with median OS of 13.5 months vs. 12.6 months (HR 0.79; 95% CI 0.60–1.04; *p* = 0.046), median PFS of 7.4 months vs. 5.5 months (HR 0.75; 95% CI 0.58–0.97; *p* = 0.0015) and overall response rate (ORR) of 41.5% vs. 15.0%, with similar safety profile in the two treatment arms [75].

### 4.2. Targeted Agents

#### 4.2.1. Fibroblast Growth Factor (FGF) Pathway Inhibitors

FGF signaling plays a pivotal role in the development, metabolism, angiogenesis and embryogenesis [76]. FGFs bind to FGFR, promoting their biological actions. There are 5 types of FGFRs: FGFR1 to 5, with FGFR5 lacking the tyrosine kinase domain [77,78]. The binding of the ligand leads to receptor dimerization, trans-auto-phosphorylation which leads to the activation of various downstream signaling pathways [79]. Altered FGF signaling is implicated in the progression of a multitude of malignancies such as breast, pancreatic cancer and melanoma [80]. Aberrant function of FGFRs has been implicated in resistance to molecularly targeted therapeutics [81].

Pemigatinib is a selective oral inhibitor of FGFR1-3 which showed effectiveness in preclinical studies [30]. In May 2020 FDA approved pemigatinib for the second-line treatment of metastatic cholangiocarcinoma patients who harbor FGFR mutations. The approval was based on the results of FIGHT-202, an open label, multicohort, single arm, phase 2 study in patients with previously treated metastatic cholangiocarcinoma. There were three cohorts: patients with *FGFR2* fusions or rearrangements (*n* = 107), patients with other *FGF/FGFR* alterations (*n* = 20) and patients with no *FGF/FGFR* alterations (*n* = 18). With a median follow up of 17.8 months 38 (36%) patients with *FGFR2* fusions or rearrangements had an objective response, complete in 3 of them. The reported DCR was 82% and restricted to *FGFR2* fusion/rearrangement carriers. Median duration of response was 7.5 months. Most common adverse event (AE) was hyperphosphatemia (60%), were arthralgias (6%) and stomatitis (5%). Additionally, serous retinal detachment due to subretinal fluid accumulation occurred in 6 (4%) of the patients, a finding that mandates careful ophthalmological monitoring in these patients [30]. Based on these promising results, pemigatinib was granted FDA approval and the follow-on FIGHT-302 clinical trial is currently recruiting patients to evaluates he efficacy of pemigatinib in the first line setting versus chemotherapy [82].

Infigratinib is an oral pan FGFR antagonist [83]. In a single arm phase II trial, 71 pretreated patients with *FGFR2* fusion received infigratinib with ORR being the primary end point [31,84,85]. ORR was 31% with DCR 84%. Median PFS was 6.8 months. Toxicity profile was similar to pemigatinib with hyperphosphatemia being the most common AE (72%).

Derazantinib is another oral FGFR inhibitor [86]. In a small phase I/II clinical trial patients with *FGFR2* fusion achieved ORR 21% with DCR 83% [87]. Further, a *post hoc* analysis of that study reported DCR 67% in a small subset of 6 patients with FGFR2 amplification or mutations [88], suggesting the opportunity to explore derazantinib efficacy in other settings besides *FGFR2* fusions. Erdafitinib on a phase II trial achieved ORR of 67% with DCR 100% [89]. Of note erdafitinib has obtained FDA approval for the treatment of urothelial cancer patients harboring *FGFR2/3* alterations [90].

Finally, novel data on irreversible FGFR inhibitors are accumulating [91]. In fact, in patients progressing after one or more lines of treatment, futibatinib (TAS-120) was recently reported to yield ORR 37%, DCR 82%, while median PFS was 7.2 months [92]. These findings are summarized in Table 1.

#### 4.2.2. IDH Inhibitors

*IDH* mutations have been implicated in the pathogenesis of various tumor types [93]. In iCCA the occurrence of *IDH1* mutations is 7–20% and for *IDH2* mutations 3% [94].

At present a number of IDH inhibitors are being tested for the treatment of iCCA: Inhibitors of IDH1 (ivosidenib), IDH2 (AG221), and pan-IDH1/2 (AG881). Results from IDH inhibitors early clinical trials showed encouraging results. In a phase I dose escalation and expansion study, ivosidenib, IDH1 inhibitor, was given to 77 patients with previously treated CCA harboring *IDH1* mutations. Four patients achieved partial response and 40 stable disease [95]. In the subsequent phase III ClarlDHy trial 185 patients with metastatic *IDH1* mutated CCA were randomly assigned to ivosidenib or placebo. Patients randomized on the ivosidenib arm achieved statistically significant better median PFS (2.7 versus 1.4 months, HR 0.37). OS was 10.8 vs. 9.7 months although crossover was permitted on progression. Most common AEs were nausea (32.1%), diarrhea (28.8%), and fatigue (23.7%) [29].

#### 4.2.3. BRAF/MEK Inhibitors

The mitogen activated protein kinase (MAPK) signal transduction cascade mediates cell growth and cellular fate and has altered regulation in a wide range of cancers, most notably melanoma and colorectal cancer, where *BRAF V600E* mutations are targeted by mutation specific therapies [96]. *BRAF* missense mutations have been identified in up to 33% of CCA [97] with the exact frequency of mutation varying depending on the specific subtype of BTC. More recent studies suggest the true mutation rate in BTC is closer to 1–5% [98] with iCCA showing the highest rate of *BRAF* mutation [8].

Combination of a MEK inhibitor (binimetinib) with chemotherapy in a phase I/II trial of untreated BTC suggested that molecular profiling of patients may be helpful in predicting response to targeted therapies but showed no improvement in PFS [99]. However, case reports of dramatic responses to combined BRAF/MEK inhibitors (dabrafenib and trametinib) exist in patients harboring *BRAF V600E* mutations [100,101], and a BTC cohort of a phase II basket trial showed that the ORR was 47% (95% CI 31–62%) to combination therapy [102]. This suggests a promising activity in the subset of patients harboring a *BRAF V600E* mutation, which requires confirmation in further studies.

#### 4.2.4. HER2 Overexpression

HER2 overexpression is also a widely targeted somatic aberration in cancers. In breast and stomach cancer evaluation of HER2 amplification in tumor tissue and subsequent treatment with HER2 targeted therapy is now standard of care [103]. Recent genetic analysis shows that HER2 overexpression is present in 4–5% of BTC, with the overexpression rate constant over subtypes [8]. Early phase trials of small number of patients have indicated that pertuzumab plus trastuzumab has activity in HER2 overexpressed and mutated BTC, refractory to chemotherapy [104]. In this study 4/11 patients had a partial response to dual HER2 targeted treatment. A case report has also indicated the efficacy of trastuzumab/lapatinib as a potential dual anti-HER2 therapy [105]. A trial is ongoing into the efficacy of trastuzumab in combination with chemotherapy (NCT03613168) with the hope that this will be an effective new treatment for a number of patients with BTC. In addition, trastuzumab deruxtecan (DS-8201), which is an antibody-drug conjugate composed of an anti-HER2 antibody and topoisomerase I inhibitor, has shown efficacy in preclinical studies of HER2 positive BTC xenograft models. This drug is therefore now being evaluated in a phase II clinical study for BTC [106].

#### 4.2.5. Regorafenib

Regorafenib is an oral multi-kinase inhibitor that predominantly targets the proteins VEGFR (1-3), PDGFR-β and FGFR1 which are involved in tumor angiogenesis and metastasis. Regorafenib is routinely used in the treatment of gastrointestinal stromal tumor (GIST), and hepatocellular carcinoma, and also has a role in the treatment of refractory colorectal cancer [107].

A number of phase II studies have demonstrated the efficacy of regorafenib in BTC. This includes observation of disease response and superior DCR in regorafenib treated patients, refractory to standard first- or second-line chemotherapy [108,109]. In addition, a recent study of second-line regorafenib versus placebo has shown a superior PFS of 3 months (95% CI 2.3–4.9) in the interventional group versus 1.5 months (95% CI 1.2–2.0) in the placebo group [110]. These studies suggest a role for the evaluation of regorafenib in phase III studies, and for identification of patients who may benefit from regorafenib.

The search for biomarkers to predict regorafenib therapy response is still under investigation. Positivity of iCCA for MALT-1 (Mucosa-associated lymphoid tissue protein 1) has been identified as one potential prognostic factor for regorafenib [111]. Raised levels of VEGF-D, IL-6 and glycoprotein 130 (GP130) have also been associated with shorter PFS and OS [109] during regorafenib therapy.

#### 4.2.6. Neurotrophic Tyrosine Receptor Kinases (NTRK) Inhibitors

Although in a small percentage of patients (Figure 2), *NTRK* gene fusions have been associated with carcinogenesis of iCCA. They primarily potentiate tumorigenesis by autophosphorylation and activating the downstream MAPK signaling pathway [112].

Importantly, NTRK inhibitors (i.e., entrectinib or larotrectinib), have shown promising results with response rate > 75% in tumors harboring *NTRK* gene fusions, regardless of histology [113], and are currently being evaluated in clinical trials including advanced BTC (NCT02576431, NCT02568267).

## 5. Conclusions

BTC are a rare and heterogeneous group of cancers arising from the biliary tract. They have historically been classified as a single disease, but over the past decade, extensive molecular characterization has shown that various genetic/epigenetic aberrations are associated with specific subgroups of BTC [23]. It is becoming increasingly clear that these aberrations may be effective therapeutic target, paving the way to the diffusion of molecularly stratified treatment approaches in BTC. *IDH1/2* mutations and *FGFR2* gene fusions are the best characterized targets with phase III and phase II evidence, supporting the respective use of IDH- and FGFR-inhibitors in treatment-experienced patients [15]. While pemigatinib successfully reached approval as the first approved targeted agent for BTC, only a small group of patients may benefit from FGFR-directed therapy and identification of novel therapeutic targets and new active agents and/or combinations is required to broaden the reach of precision anti-cancer medicine in patients with BTC [15]. A number of factors stand as significant barriers to the achievement of this goal, including wide heterogeneity of the tumor, the complexity of the microenvironment, and the development of drug resistance [1]. The results of ongoing trials are eagerly awaited and will hopefully provide new therapies leading to incremental survival benefit in patients with advanced disease [33]. Large-scale implementation of routine molecular screening, although limited by cost-effectiveness and reimbursement considerations, is a highly desirable aim in BTC in order to enable the selection of patients that could benefit from targeted approaches [1].

## Figures and Tables

**Table 1:** Efficacy results of FGFR inhibitors in intrahepatic cholangiocarcinoma harboring *FGFR* aberrations.

Parameters	Pemigatinib [30]	Infigratinib [85]	Derazantinib [87]	Erdafitinib [89]	Futibatinib [92]
Target	FGFR 1-3	FGFR 1-3	Pan-FGFR	Pan-FGFR	Pan-FGFR (irreversible)
Phase	II	II	I/II	II	II
Patient population (N)	107	71	29	11	67 (FGFR2 fusions/rearrangements)
Baseline characteristics	FGFR2 fusions/rearrangements; ≥1 prior systemic therapy	FGFR fusions; ≥1 prior systemic therapy including cisplatin intolerant	FGFR2 fusions; ≥1 prior systemic therapy or not eligible for standard chemotherapy	FGFR aberrations (7 patients with FGFR2 fusion); ≥1 prior systemic therapy; Asian patients	FGFR2 fusions/rearrangements; ≥1 prior systemic therapy
Dose (daily)	13.5 mg	125 mg	300 mg	8 mg	20 mg
Regimen	Two weeks on, one week off	Three weeks on, one week off	Continuous dosing	Continuous dosing	Continuous for 21-day cycle
Route of administration	Oral	Oral	Oral	Oral	Oral
ORR (%)	35.5 (95%CI 26.5–45.4)	31 (95% CI 20.5–43.1)	20.7 (NA)	66.7 (NA) *	37.3 (95% CI 27.8–50)
DCR (%)	82.0 (95% CI 74–89)	83.6 (95% CI 72.5–91.5)	82.8 (NA)	100 (NA) *	82.1 (95% CI 70.8–90.4)
Median DoR (months)	7.5 (95% CI 5.7–14.5)	5.4 (95% CI 3.7–7.4)	4.6 (95% CI 2.3–8.9)	3.94 (NA)	6.2 (95% CI 2.0-15.8)
Median PFS (months)	6.9 (95% CI 6.2–9.6)	6.8 (95% CI 5.3–7.6)	5.7 (95% CI 4.0–9.2)	5.59 (95% CI 1.87–NE)	7.2 (95% CI 4.9–15.2)
Median OS (months)	21.1 (95% CI 14.8–NE)	12.5 (95% CI 9.9–16.6)	Not reached	NA	NA

Abbreviations: FGFR: Fibroblast growth factor receptor; IQR: Interquartile range; NA: Not available; DoT: Duration of treatment; ORR: Overall response rate; CI: Confidence interval; DCR: Disease-control rate; DoR: Duration of response; PFS: Progression-free survival; NE: Not estimable; OS: Overall survival. * FGFR2 aberrations.

## References

[1] Rimassa L., Personeni N., Aghemo A., Lleo A. (2019). The immune milieu of cholangiocarcinoma: From molecular pathogenesis to precision medicine. J. Autoimmun..

[2] Ebata T., Ercolani G., Alvaro D., Ribero D., Di Tommaso L., Valle J.W. (2017). Current Status on Cholangiocarcinoma and Gallbladder Cancer. Liver Cancer.

[3] Patel N., Benipal B. (2019). Incidence of Cholangiocarcinoma in the USA from 2001 to 2015: A US Cancer Statistics Analysis of 50 States. Cureus.

[4] Khan S.A., Tavolari S., Brandi G. (2019). Cholangiocarcinoma: Epidemiology and risk factors. Liver Int..

[5] Bertuccio P., Malvezzi M., Carioli G., Hashim D., Boffetta P., El-Serag H.B., La Vecchia C., Negri E. (2019). Global trends in mortality from intrahepatic and extrahepatic cholangiocarcinoma. J. Hepatol..

[6] Akinyemiju T., Abera S., Ahmed M., Alam N., Alemayohu M.A., Allen C., Al-Raddadi R., Alvis-Guzman N., Amoako Y., Global Burden of Disease Liver Cancer Collaboration (2017). The Burden of Primary Liver Cancer and Underlying Etiologies From 1990 to 2015 at the Global, Regional, and National Level. JAMA Oncol..

[7] Razumilava N., Gores G.J. (2014). Cholangiocarcinoma. Lancet.

[8] Nakamura H., Arai Y., Totoki Y., Shirota T., ElZawahry A., Kato M., Hama N., Hosoda F., Urushidate T., Ohashi S. (2015). Genomic spectra of biliary tract cancer. Nat. Genet..

[9] Lamarca A., Ross P., Wasan H.S.A., Hubner R., McNamara M.G., Lopes A., Manoharan P., Palmer D., Bridgewater J., Valle J.W. (2019). Advanced intrahepatic cholangiocarcinoma: Post-hoc analysis of the ABC-01, -02 and -03 clinical trials. J. Natl. Cancer Inst..

[10] Rizvi S., Gores G.J. (2013). Pathogenesis, Diagnosis, and Management of Cholangiocarcinoma. Gastroenterology.

[11] Rizvi S., Khan S.A., Hallemeier C.L., Kelley R.K., Gores G.J. (2018). Cholangiocarcinoma—evolving concepts and therapeutic strategies. Nat. Rev. Clin. Oncol..

[12] Petrick J.L., Thistle J.E., Zeleniuch-Jacquotte A., Zhang X., Wactawski-Wende J., Van Dyke A.L., Stampfer M.J., Sinha R., Sesso H.D., Schairer C. (2018). Body Mass Index, Diabetes and Intrahepatic Cholangiocarcinoma Risk: The Liver Cancer Pooling Project and Meta-analysis. Am. J. Gastroenterol..

[13] Braconi C., Roessler S., Kruk B., Lammert F., Krawczyk M., Andersen J.B. (2019). Molecular perturbations in cholangiocarcinoma: Is it time for precision medicine?. Liver Int..

[14] Kelley R.K., Bridgewater J., Gores G.J., Zhu A.X. (2020). Systemic therapies for intrahepatic cholangiocarcinoma. J. Hepatol..

[15] Lamarca A., Barriuso J., McNamara M.G., Valle J.W. (2020). Molecular targeted therapies: Ready for “prime time” in biliary tract cancer. J. Hepatol..

[16] Hainsworth J.D., Rubin M.S., Spigel D.R., Boccia R.V., Raby S., Quinn R., Greco F.A. (2013). Molecular Gene Expression Profiling to Predict the Tissue of Origin and Direct Site-Specific Therapy in Patients With Carcinoma of Unknown Primary Site: A Prospective Trial of the Sarah Cannon Research Institute. J. Clin. Oncol..

[17] Primrose J.N., Fox R.P., Palmer D.H., Malik H.Z., Prasad R., Mirza D., Anthony A., Corrie P., Falk S., Finch-Jones M. (2019). Capecitabine compared with observation in resected biliary tract cancer (BILCAP): A randomised, controlled, multicentre, phase 3 study. Lancet Oncol..

[18] Shroff R.T., Kennedy E.B., Bachini M., Bekaii-Saab T., Crane C., Edeline J., El-Khoueiry A., Feng M., Katz M.H., Primrose J. (2019). Adjuvant Therapy for Resected Biliary Tract Cancer: ASCO Clinical Practice Guideline. J. Clin. Oncol..

[19] Valle J., Wasan H., Palmer D.H., Cunningham D., Anthoney A., Maraveyas A., Madhusudan S., Iveson T., Hughes S., Pereira S.P. (2010). Cisplatin plus Gemcitabine versus Gemcitabine for Biliary Tract Cancer. N. Engl. J. Med..

[20] Okusaka T., Nakachi K., Fukutomi A., Mizuno N., Ohkawa S., Funakoshi A., Nagino M., Kondo S., Nagaoka S., Funai J. (2010). Gemcitabine alone or in combination with cisplatin in patients with biliary tract cancer: A comparative multicentre study in Japan. Br. J. Cancer.

[21] Valle J., Furuse J., Jitlal M., Beare S., Mizuno N., Wasan H., Bridgewater J., Okusaka T. (2014). Cisplatin and gemcitabine for advanced biliary tract cancer: A meta-analysis of two randomised trials. Ann. Oncol..

[22] Lamarca A., Palmer D.H., Wasan H.S., Ross P.J., Ma Y.T., Arora A., Falk S., Gillmore R., Wadsley J., Patel K. (2019). ABC-06 | A randomised phase III, multi-centre, open-label study of active symptom control (ASC) alone or ASC with oxaliplatin/5-FU chemotherapy (ASC+mFOLFOX) for patients (pts) with locally advanced / metastatic biliary tract cancers (ABC) previously-treated with cisplatin/gemcitabine (CisGem) chemotherapy. J. Clin. Oncol..

[23] Malenica I., Donadon M., Lleo A. (2020). Molecular and Immunological Characterization of Biliary Tract Cancers: A Paradigm Shift Towards a Personalized Medicine. Cancers.

[24] Banales J.M., Marin J.J.G., Lamarca A., Rodrigues P.M., Khan S.A., Roberts L.R., Cardinale V., Carpino G., Andersen J.B., Braconi C. (2020). Cholangiocarcinoma 2020: The next horizon in mechanisms and management. Nat. Rev. Gastroenterol. Hepatol..

[25] Valle J.W., Borbath I., Khan S.A., Huguet F., Gruenberger T., Arnold D. (2016). Biliary cancer: ESMO Clinical Practice Guidelines for diagnosis, treatment and follow-up. Ann. Oncol..

[26] Forner A., Vidili G., Rengo M., Bujanda L., Ponz-Sarvisé M., Lamarca A. (2019). Clinical presentation, diagnosis and staging of cholangiocarcinoma. Liver Int..

[27] Macias R.I.R., Kornek M., Rodrigues P.M., Paiva N.A., Castro R.E., Urban S., Pereira S.P., Cadamuro M., Rupp C., Loosen S.H. (2019). Diagnostic and prognostic biomarkers in cholangiocarcinoma. Liver Int..

[28] Bailey A., Shah S.A. (2019). Screening high risk populations for cancer: Hepatobiliary. J. Surg. Oncol..

[29] Abou-Alfa G.K., Macarulla T., Javle M.M., Kelley R.K., Lubner S.J., Adeva J., Cleary J.M., Catenacci D.V., Borad M.J., Bridgewater J. (2020). Ivosidenib in IDH1-mutant, chemotherapy-refractory cholangiocarcinoma (ClarIDHy): A multicentre, randomised, double-blind, placebo-controlled, phase 3 study. Lancet Oncol..

[30] Abou-Alfa G.K., Sahai V., Hollebecque A., Vaccaro G., Melisi D., Al-Rajabi R., Paulson A.S., Borad M.J., Gallinson D., Murphy A.G. (2020). Pemigatinib for previously treated, locally advanced or metastatic cholangiocarcinoma: A multicentre, open-label, phase 2 study. Lancet Oncol..

[31] Javle M., Lowery M., Shroff R.T., Weiss K.H., Springfeld C., Borad M.J., Ramanathan R.K., Goyal L., Sadeghi S., Macarulla T. (2018). Phase II Study of BGJ398 in Patients With FGFR-Altered Advanced Cholangiocarcinoma. J. Clin. Oncol..

[32] Valle J.W., Lamarca A., Goyal L., Barriuso J., Zhu A.X. (2017). New Horizons for Precision Medicine in Biliary Tract Cancers. Cancer Discov..

[33] Adeva J., Sangro B., Salati M., Edeline J., La Casta A., Bittoni A., Berardi R., Bruix J., Valle J.W. (2019). Medical treatment for cholangiocarcinoma. Liver Int..

[34] Saha S.K., Parachoniak C.A., Ghanta K.S., Fitamant J., Ross K.N., Najem M.S., Gurumurthy S., Akbay E.A., Sia D., Cornella H. (2014). Mutant IDH inhibits HNF-4α to block hepatocyte differentiation and promote biliary cancer. Nat. Cell Biol..

[35] Lowery M.A., Ptashkin R.N., Jordan E.J., Berger M.F., Zehir A., Capanu M., Kemeny N.E., O’Reilly E.M., El-Dika I., Jarnagin W.R. (2018). Comprehensive Molecular Profiling of Intrahepatic and Extrahepatic Cholangiocarcinomas: Potential Targets for Intervention. Clin. Cancer Res..

[36] Walter D., Hartmann S., Waidmann O. (2017). Update on cholangiocarcinoma: Potential impact of genomic studies on clinical management. Z. Gastroenterol..

[37] Graham R.P., Fritcher E.G.B., Pestova E., Schulz J., Sitailo L.A., Vasmatzis G., Murphy S.J., Bamlet W.R., Hart S.N., Halling K.C. (2014). Fibroblast growth factor receptor 2 translocations in intrahepatic cholangiocarcinoma. Hum. Pathol..

[38] Hollebecque A., Silverman I., Owens S., Féliz L., Lihou C., Zhen H., Newton R., Burn T., Melisi D. (2019). Comprehensive genomic profiling and clinical outcomes in patients (pts) with fibroblast growth factor receptor rearrangement-positive (FGFR2+) cholangiocarcinoma (CCA) treated with pemigatinib in the fight-202 trial. Ann. Oncol..

[39] Farshidfar F., Zheng S., Gingras M.-C., Newton Y., Shih J.A., Robertson G., Hinoue T., Hoadley K.A., Gibb E.A., Roszik J. (2017). Integrative Genomic Analysis of Cholangiocarcinoma Identifies Distinct IDH-Mutant Molecular Profiles. Cell Rep..

[40] Sia D., Hoshida Y., Villanueva A., Roayaie S., Ferrer J., Tabak B., Peix J., Sole M., Tovar V., Alsinet C. (2013). Integrative Molecular Analysis of Intrahepatic Cholangiocarcinoma Reveals 2 Classes That Have Different Outcomes. Gastroenterology.

[41] Cao J., Hu J., Liu S., Meric-Bernstam F., Abdel-Wahab R., Xu J., Li Q., Yan M., Feng Y., Lin J. (2020). Intrahepatic Cholangiocarcinoma: Genomic Heterogeneity Between Eastern and Western Patients. JCO Precis. Oncol..

[42] Fingas C.D., Katsounas A., Kahraman A., Siffert W., Jochum C., Gerken G., Nückel H., Canbay A. (2009). Prognostic Assessment of Three Single-Nucleotide Polymorphisms (GNB3825C>T,BCL2-938C>A,MCL1-386C>G) in Extrahepatic Cholangiocarcinoma. Cancer Investig..

[43] Tella S.H., Kommalapati A., Borad M.J., Mahipal A. (2020). Second-line therapies in advanced biliary tract cancers. Lancet Oncol..

[44] Montal R., Sia D., Montironi C., Leow W.Q., Esteban-Fabró R., Pinyol R., Torres-Martin M., Bassaganyas L., Moeini A., Peix J. (2020). Molecular classification and therapeutic targets in extrahepatic cholangiocarcinoma. J. Hepatol..

[45] Bustos B.I., Pérez-Palma E., Buch S., Azócar L., Riveras E., Ugarte G.D., Toliat M., Nürnberg P., Lieb W., Franke A. (2019). Variants in ABCG8 and TRAF3 genes confer risk for gallstone disease in admixed Latinos with Mapuche Native American ancestry. Sci. Rep..

[46] Baselga J., Swain S.M. (2009). Novel anticancer targets: Revisiting ERBB2 and discovering ERBB3. Nat. Rev. Cancer.

[47] Hyman D.M., Piha-Paul S.A., Won H., Rodon J., Saura C., Shapiro G.I., Juric D., Quinn D., Moreno V., Doger B. (2018). HER kinase inhibition in patients with HER2- and HER3-mutant cancers. Nat. Cell Biol..

[48] Mhatre S., Wang Z., Nagrani R., Badwe R., Chiplunkar S., Mittal B., Yadav S., Zhang H., Chung C.C., Patil P. (2017). Common genetic variation and risk of gallbladder cancer in India: A case-control genome-wide association study. Lancet Oncol..

[49] Fujimoto A., Furuta M., Shiraishi Y., Gotoh K., Kawakami Y., Arihiro K., Nakamura T., Ueno M., Ariizumi S.-I., Nguyen H.H. (2015). Whole-genome mutational landscape of liver cancers displaying biliary phenotype reveals hepatitis impact and molecular diversity. Nat. Commun..

[50] Feinberg A.P., Ohlsson R., Henikoff S. (2006). The epigenetic progenitor origin of human cancer. Nat. Rev. Genet..

[51] Yang B., House M.G., Guo M., Herman J.G., Clark D.P. (2004). Promoter methylation profiles of tumor suppressor genes in intrahepatic and extrahepatic cholangiocarcinoma. Mod. Pathol..

[52] Jiao Y., Pawlik T.M.A., Anders R., Selaru F.M., Streppel M.M., Lucas D.J., Niknafs N., Guthrie V.B., Maitra A., Argani P. (2013). Exome sequencing identifies frequent inactivating mutations in BAP1, ARID1A and PBRM1 in intrahepatic cholangiocarcinomas. Nat. Genet..

[53] Goeppert B., Konermann C., Schmidt C.R., Bogatyrova O., Geiselhart L., Ernst C., Gu L., Becker N., Zucknick M., Mehrabi A. (2014). Global alterations of DNA methylation in cholangiocarcinoma target the Wnt signaling pathway. Hepatology.

[54] O’Rourke C.J., Munoz-Garrido P., Aguayo E.L., Andersen J.B. (2018). Epigenome dysregulation in cholangiocarcinoma. Biochim. Biophys. Acta BBA Mol. Basis Dis..

[55] Yu H., Pak H., Hammond-Martel I., Ghram M., Rodrigue A., Daou S., Barbour H., Corbeil L., Hébert J., Drobetsky E. (2013). Tumor suppressor and deubiquitinase BAP1 promotes DNA double-strand break repair. Proc. Natl. Acad. Sci. USA.

[56] Shen J., Peng Y., Wei L., Zhang W., Yang L., Lan L., Kapoor P., Ju Z., Mo Q., Shih I.-M. (2015). ARID1A Deficiency Impairs the DNA Damage Checkpoint and Sensitizes Cells to PARP Inhibitors. Cancer Discov..

[57] Nakagawa S., Okabe H., Sakamoto Y., Hayashi H., Hashimoto D., Yokoyama N., Sakamoto K., Kuroki H., Mima K., Nitta H. (2013). Enhancer of Zeste Homolog 2 (EZH2) Promotes Progression of Cholangiocarcinoma Cells by Regulating Cell Cycle and Apoptosis. Ann. Surg. Oncol..

[58] Turcan S., Rohle D., Goenka A., Walsh L.A., Fang F., Yilmaz E., Campos C., Fabius A.W.M., Lu C., Ward P.S. (2012). IDH1 mutation is sufficient to establish the glioma hypermethylator phenotype. Nat. Cell Biol..

[59] Wang P., Dong Q., Zhang C., Kuan P.-F., Liu Y., Jeck W.R., Andersen J.B., Jiang W., Savich G.L., Tan T.-X. (2013). Mutations in isocitrate dehydrogenase 1 and 2 occur frequently in intrahepatic cholangiocarcinomas and share hypermethylation targets with glioblastomas. Oncogene.

[60] De Botton S., Mondesir J., Willekens C., Touat M. (2016). IDH1 and IDH2 mutations as novel therapeutic targets: Current perspectives. J. Blood Med..

[61] Boscoe A.N., Rolland C., Kelley R.K. (2019). Frequency and prognostic significance of isocitrate dehydrogenase 1 mutations in cholangiocarcinoma: A systematic literature review. J. Gastrointest. Oncol..

[62] Dang L., Su S.-S.M. (2017). Isocitrate Dehydrogenase Mutation and (R)-2-Hydroxyglutarate: From Basic Discovery to Therapeutics Development. Annu. Rev. Biochem..

[63] Peraldo-Neia C., Ostano P., Cavalloni G., Pignochino Y., Sangiolo D., De Cecco L., Marchesi E., Ribero D., Scarpa A., De Rose A.M. (2018). Transcriptomic analysis and mutational status of IDH1 in paired primary-recurrent intrahepatic cholangiocarcinoma. BMC Genom..

[64] Goeppert B., Toth R., Singer S., Albrecht T., Lipka D.B., Lutsik P., Brocks D., Baehr M., Muecke O., Assenov Y. (2019). Integrative Analysis Defines Distinct Prognostic Subgroups of Intrahepatic Cholangiocarcinoma. Hepatology.

[65] Zou S., Li J., Zhou H., Frech C., Jiang X., Chu J.S.C., Zhao X., Li Y., Li Q., Wang H. (2014). Mutational landscape of intrahepatic cholangiocarcinoma. Nat. Commun..

[66] Jusakul A., Cutcutache I., Yong C.H., Lim J.Q., Ni Huang M., Padmanabhan N., Nellore V., Kongpetch S., Ng A.W.T., Ng L.M. (2017). Whole-Genome and Epigenomic Landscapes of Etiologically Distinct Subtypes of Cholangiocarcinoma. Cancer Discov..

[67] Oishi N., Kumar M.R., Roessler S., Ji J., Forgues M., Budhu A., Zhao X., Andersen J.B., Ye Q.-H., Jia H.-L. (2012). Transcriptomic profiling reveals hepatic stem-like gene signatures and interplay of miR-200c and epithelial-mesenchymal transition in intrahepatic cholangiocarcinoma. Hepatology.

[68] Glimelius B., Hoffman K., Sjödén P.-O., Jacobsson G., Sellström H., Enander L.-K., Linné T., Svensson C. (1996). Chemotherapy improves survival and quality of life in advanced pancreatic and biliary cancer. Ann. Oncol..

[69] Eckel F., Schmidt G. (2014). Chemotherapy and Targeted Therapy in Advanced Biliary Tract Carcinoma: A Pooled Analysis of Clinical Trials. Chemotherapy.

[70] Valle J.W. (2010). Advances in the treatment of metastatic or unresectable biliary tract cancer. Ann. Oncol..

[71] Lamarca A., Benafif S., Ross P., Bridgewater J., Valle J.W. (2015). Cisplatin and gemcitabine in patients with advanced biliary tract cancer (ABC) and persistent jaundice despite optimal stenting: Effective intervention in patients with luminal disease. Eur. J. Cancer.

[72] André T., Reyes-Vidal J.M., Fartoux L., Ross P., Leslie M., Rosmorduc O., Clemens M.R., Louvet C., Perez N., Mehmud F. (2008). Gemcitabine and oxaliplatin in advanced biliary tract carcinoma: A phase II study. Br. J. Cancer.

[73] Morizane C., Okusaka T., Mizusawa J., Katayama H., Ueno M., Ikeda M., Ozaka M., Okano N., Sugimori K., Fukutomi A. (2019). Combination gemcitabine plus S-1 versus gemcitabine plus cisplatin for advanced/recurrent biliary tract cancer: The FUGA-BT (JCOG1113) randomized phase III clinical trial. Ann. Oncol..

[74] Shroff R.T., Javle M.M., Xiao L., Kaseb A.O., Varadhachary G.R., Wolff R.A., Raghav K.P.S., Iwasaki M., Masci P., Ramanathan R.K. (2019). Gemcitabine, Cisplatin, and nab-Paclitaxel for the Treatment of Advanced Biliary Tract Cancers. JAMA Oncol..

[75] Sakai D., Kanai M., Kobayashi S., Eguchi H., Baba H., Seo S., Taketomi A., Takayama T., Yamaue H., Ishioka C. (2018). Randomized phase III study of gemcitabine, cisplatin plus S-1 (GCS) versus gemcitabine, cisplatin (GC) for advanced biliary tract cancer (KHBO1401-MITSUBA). Ann. Oncol..

[76] Belov A.A., Mohammadi M. (2013). Molecular Mechanisms of Fibroblast Growth Factor Signaling in Physiology and Pathology. Cold Spring Harb. Perspect. Biol..

[77] Boöttcher R.T., Niehrs C. (2004). Fibroblast Growth Factor Signaling during Early Vertebrate Development. Endocr. Rev..

[78] Sleeman M., Fraser J., McDonald M., Yuan S., White D., Grandison P., Kumble K., Watson J.D., Murison J. (2001). Identification of a new fibroblast growth factor receptor, FGFR5. Gene.

[79] Sarabipour S., Hristova K. (2016). Mechanism of FGF receptor dimerization and activation. Nat. Commun..

[80] Hallinan N., Finn S., Cuffe S., Rafee S., O’Byrne K., Gately K. (2016). Targeting the fibroblast growth factor receptor family in cancer. Cancer Treat. Rev..

[81] Wang V.E., Xue Y., Frederick D.T., Cao Y., Lin E., Wilson C., Urisman A., Carbone D.P., Flaherty K.T., Bernards R. (2019). Adaptive Resistance to Dual BRAF/MEK Inhibition in BRAF-Driven Tumors through Autocrine FGFR Pathway Activation. Clin. Cancer Res..

[82] Bekaii-Saab T.S., Valle J.W., Van Cutsem E., Rimassa L., Furuse J., Ioka T., Melisi D., Macarulla T., Bridgewater J., Wasan H. (2020). FIGHT-302: First-line pemigatinib vs gemcitabine plus cisplatin for advanced cholangiocarcinoma with FGFR2 rearrangements. Futur. Oncol..

[83] Guagnano V., Kauffmann A., Wöhrle S., Stamm C., Ito M., Barys L., Pornon A., Yao Y., Li F., Zhang Y. (2012). FGFR Genetic Alterations Predict for Sensitivity to NVP-BGJ398, a Selective Pan-FGFR Inhibitor. Cancer Discov..

[84] Javle M., Kelley R.K., Roychowdhury S., Weiss K.H., Abou-Alfa G.K., Macarulla T., Sadeghi S., Waldschmidt D., Zhu A.X., Goyal L. (2019). AB051. P-19. A phase II study of infigratinib (BGJ398) in previously-treated advanced cholangiocarcinoma containing FGFR2 fusions. HepatoBiliary Surg. Nutr..

[85] Javle M., Kelley R., Roychowdhury S., Weiss K., Abou-Alfa G., Macarulla T., Sadeghi S., Waldschmidt D., Zhu A., Goyal L. (2018). Updated results from a phase II study of infigratinib (BGJ398), a selective pan-FGFR kinase inhibitor, in patients with previously treated advanced cholangiocarcinoma containing FGFR2 fusions. Ann. Oncol..

[86] Hall T.G., Yu Y., Eathiraj S., Wang Y., Savage R.E., Lapierre J.-M., Schwartz B., Abbadessa G. (2016). Preclinical Activity of ARQ 087, a Novel Inhibitor Targeting FGFR Dysregulation. PLoS ONE.

[87] Mazzaferro V., El-Rayes B., Busset M.D.D., Cotsoglou C., Harris W.P., Damjanov N., Masi G., Rimassa L., Personeni N., Braiteh F. (2019). Derazantinib (ARQ 087) in advanced or inoperable FGFR2 gene fusion-positive intrahepatic cholangiocarcinoma. Br. J. Cancer.

[88] Busset M.D.D., Braun S., El-Rayes B., Harris W., Damjanov N., Masi G., Rimassa L., Bhoori S., Niger M., Personeni N. (2019). Efficacy of derazantinib (DZB) in patients (pts) with intrahepatic cholangiocarcinoma (iCCA) expressing FGFR2-fusion or FGFR2 mutations/amplifications. Ann. Oncol..

[89] Chen Y.-Y., Park J., Su W.-C., Oh D.-Y., Kim K.-P., Feng Y.-H., Shen L., Liao H., Nie J., Qing M. (2018). Preliminary results of a ph2a study to evaluate the clinical efficacy and safety of erdafitinib in Asian patients with biomarker-selected advanced cholangiocarcinoma (CCA). Ann. Oncol..

[90] Loriot Y., Necchi A., Park S.H., Garcia-Donas J., Huddart R., Burgess E., Fleming M., Rezazadeh A., Mellado B., Varlamov S. (2019). Erdafitinib in Locally Advanced or Metastatic Urothelial Carcinoma. N. Engl. J. Med..

[91] Sootome H., Fujita H., Ito K., Ochiiwa H., Fujioka Y., Ito K., Miura A., Sagara T., Ito S., Ohsawa H. (2020). Futibatinib is a novel irreversible FGFR 1-4 inhibitor that shows selective antitumor activity against FGFR-deregulated tumors. Cancer Res..

[92] Goyal L., Meric-Bernstam F., Hollebecque A., Valle J.W., Morizane C., Karasic T.B., Abrams T.A., Furuse J., He Y., Soni N. (2020). FOENIX-CCA2: A phase II, open-label, multicenter study of futibatinib in patients (pts) with intrahepatic cholangiocarcinoma (iCCA) harboring FGFR2 gene fusions or other rearrangements. J. Clin. Oncol..

[93] Al-Khallaf H. (2017). Isocitrate dehydrogenases in physiology and cancer: Biochemical and molecular insight. Cell Biosci..

[94] Romanidou O., Kotoula V., Fountzilas G. (2018). Bridging Cancer Biology with the Clinic: Comprehending and Exploiting IDH Gene Mutations in Gliomas. Cancer Genom. Proteom..

[95] Lowery M.A., Abou-Alfa G.K., Burris H.A., Janku F., Shroff R.T., Cleary J.M., Azad N.S., Goyal L., Maher E.A., Gore L. (2017). Phase I study of AG-120, an IDH1 mutant enzyme inhibitor: Results from the cholangiocarcinoma dose escalation and expansion cohorts. J. Clin. Oncol..

[96] Ross J.S., Wang K., Chmielecki J., Gay L., Johnson A., Chudnovsky J., Yelensky R., Lipson D., Ali S.M., Elvin J.A. (2016). The distribution of BRAF gene fusions in solid tumors and response to targeted therapy. Int. J. Cancer.

[97] Morizane C., Ueno M., Ikeda M., Okusaka T., Ishii H., Furuse J. (2018). New developments in systemic therapy for advanced biliary tract cancer. Jpn. J. Clin. Oncol..

[98] Chun Y.S., Javle M. (2017). Systemic and Adjuvant Therapies for Intrahepatic Cholangiocarcinoma. Cancer Control..

[99] Lowery M.A., Bradley M., Chou J.F., Capanu M., Gerst S., Harding J.J., El Dika I., Berger M., Zehir A., Ptashkin R. (2019). Binimetinib plus Gemcitabine and Cisplatin Phase I/II Trial in Patients with Advanced Biliary Cancers. Clin. Cancer Res..

[100] Lavingia V., Fakih M. (2016). Impressive response to dual BRAF and MEK inhibition in patients with BRAF mutant intrahepatic cholangiocarcinoma—2 case reports and a brief review. J. Gastrointest. Oncol..

[101] Tannapfel A., Sommerer F., Benicke M., Katalinic A., Uhlmann D., Witzigmann H., Hauss J., Wittekind C. (2003). Mutations of the BRAF gene in cholangiocarcinoma but not in hepatocellular carcinoma. Gut.

[102] Subbiah V., Lassen U., Élez E., Italiano A., Curigliano G., Javle M., De Braud F., Prager G.W., Greil R., Stein A. (2020). Dabrafenib plus trametinib in patients with BRAFV600E-mutated biliary tract cancer (ROAR): A phase 2, open-label, single-arm, multicentre basket trial. Lancet Oncol..

[103] Meric-Bernstam F., Johnson A.M., Dumbrava E.E.I., Raghav K., Balaji K., Bhatt M., Murthy R.K., Rodon J., Piha-Paul S.A. (2018). Advances in HER2-Targeted Therapy: Novel Agents and Opportunities Beyond Breast and Gastric Cancer. Clin. Cancer Res..

[104] Javle M., Hainsworth J.D., Swanton C., Burris H.A., Kurzrock R., Sweeney C., Meric-Bernstam F., Spigel D.R., Bose R., Guo S. (2017). Pertuzumab + trastuzumab for HER2-positive metastatic biliary cancer: Preliminary data from MyPathway. J. Clin. Oncol..

[105] Yarlagadda B., Kamatham V., Ritter A., Shahjehan F., Kasi P.M. (2019). Trastuzumab and pertuzumab in circulating tumor DNA ERBB2-amplified HER2-positive refractory cholangiocarcinoma. NPJ Precis. Oncol..

[106] Ohba A., Morizane C., Ueno M., Kobayashi S., Kawamoto Y., Komatsu Y., Ikeda M., Sasaki M., Okano N., Furuse J. (2020). Multicenter phase II study of trastuzumab deruxtecan (DS-8201) for HER2-positive unresectable or recurrent biliary tract cancer: HERB trial. J. Clin. Oncol..

[107] Grothey A., Blay J.-Y., Pavlakis N., Yoshino T., Bruix J. (2020). Evolving role of regorafenib for the treatment of advanced cancers. Cancer Treat. Rev..

[108] Sun W., Patel A., Normolle D.P., Patel K., Ohr J., Lee J.J., Bahary N., Chu E., Streeter N., Drummond S. (2019). A phase 2 trial of regorafenib as a single agent in patients with chemotherapy-refractory, advanced, and metastatic biliary tract adenocarcinoma. Cancer.

[109] Kim R., Sanoff H.K., Poklepovic A., Soares H., Kim J., Lyu J., Liu Y., Nixon A.B., Kim D.W. (2020). A multi-institutional phase 2 trial of regorafenib in refractory advanced biliary tract cancer. Cancer.

[110] Demols A., Borbath I., Eynde M.V.D., Houbiers G., Peeters M., Marechal R., Delaunoit T., Goemine J.-C., Laurent S., Holbrechts S. (2020). Regorafenib after failure of gemcitabine and platinum-based chemotherapy for locally advanced/metastatic biliary tumors: REACHIN, a randomized, double-blind, phase II trial. Ann. Oncol..

[111] Yeh C.-N., Chang Y.-C., Su Y., Hsu D.S.-S., Cheng C.-T., Wu R.-C., Chung Y.-H., Chiang K.-C., Yeh T.-S., Lu M.-L. (2017). Identification of MALT1 as both a prognostic factor and a potential therapeutic target of regorafenib in cholangiocarcinoma patients. Oncotarget.

[112] Ross J.S., Wang K., Gay L., Al-Rohil R., Rand J.V., Jones D.M., Lee H.J., Sheehan C.E., Otto G.A., Palmer G. (2014). New Routes to Targeted Therapy of Intrahepatic Cholangiocarcinomas Revealed by Next-Generation Sequencing. Oncologist.

[113] Cocco E., Scaltriti M., Drilon A. (2018). NTRK fusion-positive cancers and TRK inhibitor therapy. Nat. Rev. Clin. Oncol..

